# Apolipoprotein B and angiotensin-converting enzyme polymorphisms and aerobic interval training: randomized controlled trial in coronary artery disease patients

**DOI:** 10.1590/1414-431X20186944

**Published:** 2018-05-28

**Authors:** N.Y. Tamburus, R. Verlengia, V.C. Kunz, M.C. César, E. Silva

**Affiliations:** 1 Núcleo de Pesquisa em Exercício Físico, Departamento de Fisioterapia, Universidade Federal de São Carlos, São Carlos, SP, Brasil; 2 Laboratório de Performance Humana, Faculdade de Ciências da Saúde, Universidade Metodista de Piracicaba, Piracicaba, SP, Brasil; 3 Centro Universitário Adventista de São Paulo, Engenheiro Coelho, SP, Brasil

**Keywords:** Physical training, Lipids, Apolipoprotein B polymorphism, Angiotensin-converting enzyme polymorphism, Aerobic capacity

## Abstract

Physical training has been strongly recommended as a non-pharmacological treatment for coronary artery disease (CAD). Genetic polymorphisms have been studied to understand the biological variability in response to exercise among individuals. This study aimed to verify the possible influence of apolipoprotein B (*ApoB*: rs1042031 and rs693) and angiotensin-converting enzyme (*ACE-ID*: rs1799752) genotypes on the lipid profile and functional aerobic capacity, respectively, after an aerobic interval training (AIT) program in patients with CAD and/or cardiovascular risk factors. Sixty-six men were randomized and assigned to trained group (n=32) or control group (n=34). Cardiopulmonary exercise test was performed to determine the ventilatory anaerobic threshold (VAT) from cardiorespiratory variables. The AIT program, at an intensity equivalent to %VAT (70–110%), was conducted three times a week for 16 weeks. *ApoB* gene polymorphisms (−12669C>T (rs1042031) and −7673G>A (rs693)) were identified by real-time polymerase chain reaction (PCR). I/D polymorphism in the *ACE* gene (rs1799752) was identified through PCR and fragment size analysis. After 16 weeks, low-density lipoprotein (LDL) levels increased in the trained and control groups with the GA+AA genotype (−7673G>A) of the *ApoB* gene. Trained groups with *ACE-II* and *ACE-ID* genotypes presented an increase in oxygen consumption (VO_2VAT_) and power output after the AIT program. The presence of the ACE I-allele was associated with increased aerobic functional capacity after the AIT program. Increased LDL levels were observed over time in patients with the −7673G>A polymorphism of the *ApoB* gene. Trial Registration Information: ClinicalTrials.gov: NCT02313831

## Introduction

Coronary artery disease (CAD) is a multifactorial disease influenced by a wide range of modifiable risk factors (dyslipidemia, hypertension, obesity, diabetes, and physical inactivity) as well as non-modifiable factors (age and genetic profile) (1). Exercise practice has been strongly recommended for the management of CAD and control of modifiable risk factors in the context of cardiac rehabilitation, given the benefits on the cardiovascular system and control of modifiable risk factors (2). Randomized controlled trials and meta-analyses have shown that aerobic interval training (AIT) is recommended for this population due to its effectiveness in improving aerobic functional capacity compared to continuous and moderate physical training (3–5). However, changes in serum lipids are small and have a limited effect on low-density lipoproteins (LDL), putting to question the effects of exercise training on lipid abnormalities (6,7). This inter-individual variability in adaptive responses and change in the lipid profile with physical training may be associated with genetic polymorphisms, which may alter cardiovascular and metabolic adaptations to exercise. Consequently, personalized medicine and inter-individual differences in response to an exercise training program have received increased scientific interest.

Among several single nucleotide polymorphisms (SNP), two genes that might play a role in regulating the physical function and indirectly impact the lipid profile are the angiotensin-converting enzyme (*ACE*) and apolipoprotein B (*ApoB*) polymorphisms, respectively. Particularly, LDLs are atherogenic and their levels depend on genetics and lifestyle factors such as diet and exercise (8). When expressed, the *ApoB* gene has been linked to concentrations of serum lipids in CAD. SNP rs1042031 (−12669C>T – nucleotide substitution of cytosine for thymine), located at exon 29 of the *ApoB* gene, and SNP rs693 (−7376G>A – nucleotide substitution of guanine for adenine), located at exon 26, have an additive effect on total cholesterol, apoliprotein B, triglycerides, and LDL levels in different populations (9,10).

The *ACE* gene is expressed in many tissues, such as in skeletal muscle and cardiovascular system. *ACE* insertion/deletion (*I/D*) of 287 base pairs in intron 16 – rs1799752 polymorphism has been associated with improvements in performance and exercise duration in different populations (11–14). The presence of the I-allele has resulted in lower ACE activity in blood serum and a reduced expression of the *ACE* gene transcript and capacity for angiotensin II production (15). Subjects carrying the D allele have approximately 30% (ACE I/D) and 60% (*ACE-DD*) higher serum and tissue ACE activity, and more serum angiotensin I is converted via ACE into angiotensin II in relation to subjects with the ACE-II genotype (16). The findings highlight the important role of the *ACE* I- allele in the improvement of aerobic performance after a physical training program (11,14,17). Meanwhile, the *ACE* D-allele has been associated with increased muscle strength (13,14,18) and anaerobic performance (12).

In this context, it was hypothesized that the significant changes in the lipid profile are related to polymorphisms of the *ApoB* gene (rs1042031 and rs693), and the improvement of the aerobic functional capacity is associated with the *ACE* I-allele in patients with CAD and/or cardiovascular risk factors submitted to the AIT program. Therefore, the aim of this study was to verify the possible influence of *ApoB* and *ACE* genotypes on the lipid profile and functional aerobic capacity, respectively, after the AIT program in patients with CAD and/or cardiovascular risk factors.

## Material and Methods

### Study design and participants

The present study was a randomized controlled clinical trial conducted between February 2011 and May 2014 at the Federal University of São Paulo and Methodist University of Piracicaba, São Paulo, Brazil.

A sample of 149 male patients was recruited at the Hemodynamics Center of the local Hospital. Sixty-eight patients (34 patients with stable CAD and 34 without CAD), originally from the southeastern region of Brazil, were considered eligible for this study.

All patients met three or more inclusion criteria, such as: myocardial infarction (MI) in the previous 6 months, percutaneous coronary intervention and coronary artery bypass graft in the previous 3 months, obesity (body mass index >30 kg/m^2^), sedentary lifestyle according to the International Physical Activity Questionnaire version 6, hypertension, diabetes mellitus (type 2 – non-users of insulin), and dyslipidemia. The exclusion criteria consisted of severe cardiac arrhythmias, chronic obstructive pulmonary disease, unstable angina, osteomuscular disorders, diabetes mellitus (insulin users), renal failure, sequelae associated with stroke, chest pain, and incapability to perform the cardiopulmonary exercise test (CPET) until the ventilatory anaerobic threshold (VAT).

Patients were randomly assigned into trained (n=34) or control group (n=34). Randomization sequence with a 1:1 allocation ratio was performed by an independent researcher who was not involved in the recruitment of participants. Allocation codes were concealed in sequentially numbered, sealed, opaque envelopes by the same investigator who created the randomization sequence.

All participants signed a written consent form prior to participating in the study, which was approved by the Ethics Committee of the Universidade Metodista de Piracicaba, Brazil (Protocol 04/09). The study was registered on clinicaltrials.gov with the identification code NCT02313831. The authors confirm that all ongoing and related trials for this intervention are registered.

### Measurements

Measurements were made at baseline and after 16 weeks of treatment. Prior to the start of the AIT program, patients were required to attend the laboratory on two occasions. The first visit included height and body mass assessments. In this visit, all subjects were familiarized with the equipment and experimental protocol to be used to reduce anxiety. Subjects were instructed to avoid the use of stimulants (coffee, tea, soft drinks) and alcoholic beverages 24 h prior to the tests to avoid exhaustion. The second visit included venous blood sample collection. All participants were then interviewed and examined before submaximal CPET to check health status and to confirm compliance with previously given instructions.

The assessments described above were collected prior to and 48 h after the last day of the AIT program. Controls were retested 16 weeks after the completion of baseline testing.

### Lipid profile

After an overnight 12-h fasting period, venous blood samples were collected to analyze the following parameters: total cholesterol (using the autoanalyzer method), high- and low-density lipoprotein (HDL and LDL) cholesterol (using enzymatic colorimetry), triglycerides (using automated enzymatic methods), and apolipoprotein A1 and B (nephelometry).

### Cardiopulmonary exercise test (CPET)

Functional capacity and power output were assessed by submaximal CPET, using a ramp protocol on a cycle ergometer with electromagnetic brake (LODE BV, Corival V2, Netherlands). All tests were performed in the morning. The room temperature of the testing laboratory was kept at 23°C and the relative air humidity between 40 and 60%.

The test started with 1 min of baseline recording followed by 4 min of unloaded warm-up. Intensity was individually increased according to formula proposed by Wasserman et al. (19) (power output (W) = [(height-age) × 20] – [150 + (6 × body mass)] / 100). For all patients, CPET was interrupted when submaximal heart rate (HR), obtained by Karvonen's formula (85% of maximum HR, attenuated by beta-blocker dose), was reached or surpassed the VAT level visually determined. Ventilatory and metabolic measurements were obtained on a breath-by-breath basis using a metabolic analyzer (CPX-D, Medical Graphics, USA). Electrocardiograms and HR were continuously recorded throughout the test using a 12-lead electrocardiogram (Welch Allyn CardioPerfect Workstation, USA), and blood pressure (BP) was measured manually.

VAT was determined from the loss of parallelism between oxygen uptake (VO_2_) and carbon dioxide production (VCO_2_) by three properly trained observers, as previously described by Zamunér et al. (20) and Higa et al. (21).

### Genotyping

Genomic DNA was isolated from EDTA-treated peripheral blood leukocyte cells using Illustra blood genomicPrep Mini Spin kit (GE HealthCare, USA) according to the manufacturer's instructions. Polymorphisms of the *ApoB* gene were determined by real-time PCR (ABI 7500 fast, Applied Biosystems, USA), using the TaqMan Universal PCR Master Mix (Applied Biosystems, USA), genomic DNA template (20 ng), and specific TaqMan probe assay [*APOB*: rs1042031 (−12669C>T; assay ID: C___7615381_20) and rs693 (−7673G>A; assay ID: C___7615420_20)]. Each probe was labeled with a different fluorophore (Vic or Fam, Figure S1). Vic dye is linked to the 5′-end of allele 1 probe (reporter) indicating homozygosity for alleles “C” in rs1042031 (−12669C>T) and “A” in rs693 (−7673G>A). Fam dye is linked to the allele 2 probe indicating homozygosity for alleles “T” in rs1042031 (−12669C>T) and “G” in rs693 (−7673G>A). The presence of both fluorescent signals indicated heterozygosity. The presence of a non-fluorescent quencher at the 3′-end of the probe allows for the detection of the dye fluorescence reporter with greater sensitivity.

The *ACE* (rs1799752) polymorphism was determined by PCR and fragment analysis as previously described by Verlengia et al. (22). PCR primer sequences were: sense 5′-CTG GAG ACC ACT CCC ATC CTT TCT-3′ and anti-sense 5′-GAT GTG GCC ATC ACA TTC AGA T-3′. PCR assays were carried out in a thermocycler (T-Gradient, Whatman Biometra, Germany) and *ACE* polymorphism fragments were visualized using 1.5% agarose gel after electrophoresis. The second PCR assay was performed in samples initially classified as DD in order to avoid misclassification of ID samples. In this step, sequences of PCR primers were: sense 5′-CTG GAG ACC ACT CCC ATC CTT TCT-3′ and anti-sense 5′- GAT GTG GCC ATC ACA TTC GTC AGAT-3′. To determine reproducibility and quality control, 10% of samples were randomly re-evaluated. All genotyping was performed by the same researcher who was blinded to subject data.

### Aerobic interval training program

The AIT program was individualized and administered three times per week (on alternate days) for 16 weeks. Exercise intensities were considered moderate and prescribed according to VAT (70, 80, 100, and 110% of power output reached at VAT) (23,24). Each exercise session lasted about 60 min and comprised the following steps: 1) Warm-up (10 min): stretching and low-intensity exercises (walking); 2) Exercise protocol (30–40 min): patients performed exercise training on a stationary cycle ergometer, and this period was divided into 6 steps. Step 1: 5 min at moderate intensity with the aim of reaching 80% of power output reached at VAT; Step 2 and 4: 5 min progressing up to 10 min at moderate intensity with the aim of reaching 100% of power output reached at VAT; Step 3 and 5: 5 min at moderate intensity with the aim of reaching 110% of power output reached at VAT; Step 6: 5 min at moderate intensity with the aim of reaching 70% of power output reached at VAT. The Borg CR-10 scale was used to measure the perceived exertion rate after each step. 3) Cool-down (10 min): consisted of stretching and respiratory exercises to allow BP and HR to return to near-basal values. During exercise training sessions, BP was measured by the auscultatory method and HR was monitored with intermittent verification by HR monitors (Polar Team System, Finland) to ensure that patients exercised at the target training intensity.

Subjects assigned to the control group were told to continue their daily life activities. They were contacted by telephone every month to check if there had been changes in medication and if new cardiac events had occurred. All subjects were instructed to maintain their usual diet without standardized caloric restriction.

Exercise intensity was adjusted on a monthly basis, according to methodology proposed by Sirol et al. (23) and Pithon et al. (24) by determination of the anaerobic threshold from the HR response.

### Statistical analysis

After genotype analyses, patients were divided into three groups according to genotype: −12669C>T (rs1042031) (CC, CT or TT group); 7376G>A (rs693) (CC, GA or AA group); I/D (rs1799752) (II, ID, and DD). Allele frequencies and genotype distributions were determined by directly counting the alleles, and a Hardy-Weinberg equilibrium was verified using the chi-squared test with Arlequin v3.11 software, which applies the expectation-maximization algorithm (25). *t*-tests were used to compare age and hemodynamic variables and the chi-squared test was applied for categorical variables. A two-way ANOVA (group × time), repeated measures followed by a *post hoc* Bonferroni test, was used to compare the differences between the anthropometric, lipid profile, and cardiorespiratory variables. Three-way ANOVA (group × time × genotype) was used to compare anthropometric and hemodynamic variables at rest, as well as metabolic and cardiorespiratory variables. When significant interaction was observed, the main effects were not considered and multiple comparisons were performed. The relationship between cardiorespiratory variables and *ACE* genotypes was analyzed using the Pearson correlation coefficient. Correlation coefficients were interpreted as follows: r=0.4 to 0.5 weak correlation; r=0.6 to 0.7 moderate correlation; r≥0.8 strong correlation).

## Results

**Figure 1.**
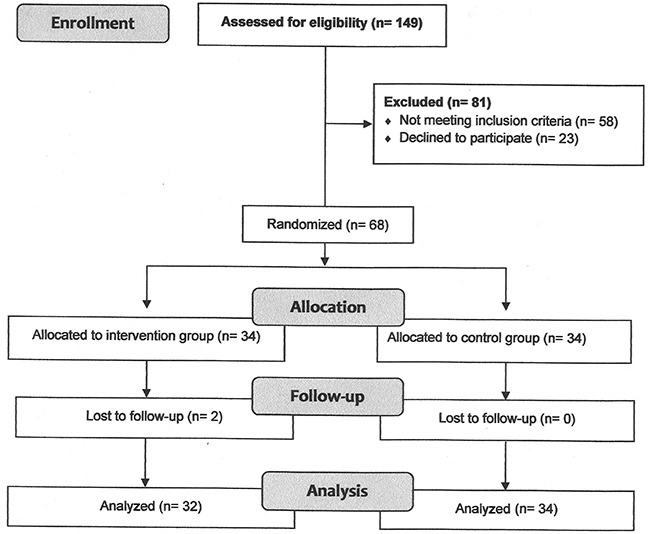
Flow chart showing the participation of patients in the study.

Figure 1 shows the study flow diagram. Initially, 149 patients were interviewed. Of these, 81 patients did not meet the inclusion criteria or refused to participate, and after randomization, two patients dropped out of the intervention program. Thus, 66 patients completed all study stages.

Table 1 presents age, anthropometric, and hemodynamic variables, cardiac events, number of diseased vessels, medications, risk factors, and allele frequencies of study groups. There were no differences between groups for any variables under study (P>0.05). The dosage of medications remained the same during the study. The observed genotype frequencies were in agreement with the Hardy-Weinberg equilibrium in both the trained group (−12669C>T *χ* ^2^=0.05, P=0.81; −7376G>A *χ* ^2^=0.008, P=0.92; *ACE I/D χ* ^2^=2.33, P=0.12) and control group (−1266C>T *χ* ^2^=0.003, P=0.95; −7376G>A *χ* ^2^=0.003, P=0.84; *ACE I/D χ* ^2^=0.0005, P=0.98). The genotypes and allele frequencies of *ApoB* and *ACE* genes did not differ between trained and control groups (P>0.05).

**Table 1.** Anthropometric and hemodynamic variables, cardiac events, number of diseased vessels, medications, risk factors, and allele frequencies of groups studied at baseline.

	Trained (n=32)	Control (n=34)	P
Age (years)	57.84±5.84	55.00±7.28	0.08
Height (cm)	82.66±14.89	82.18±12.31	0.21
Weight (kg)	169.63±7.01	169.97±6.21	0.70
SBP (mmHg)	129.78±14.08	126.12±13.99	0.29
DBP (mmHg)	84.25±9.46	81.54±8.35	0.22
HR (bpm)	64.22±10.94	65.12±12.64	0.75
Cardiac events (number of patients)			
MI	7	14	0.15
CABG	4	1	0.19
PCI	14	17	0.79
No. of diseased vessels (number of patients)			
One diseased vessel	4	2	0.42
Two diseased vessels	2	4	0.67
Three or four diseased vessels	9	10	0.87
Medication (number of patients)			
Beta blockers	16	23	0.22
ACE inhibitors	20	23	0.85
Lipid-lowering drugs	22	23	0.86
Diuretics	6	5	0.91
Antiplatelet agents	21	26	0.48
Hypoglycemic agents	3	7	0.30
Risk factor (number of patients)			
Currently smoking	5	7	0.75
Dyslipidemia	24	25	0.88
Hypertension	22	22	0.93
Overweight / obesity	21	25	0.66
Diabetes mellitus	5	7	0.75
Allele frequencies			
−7673G>A (rs603) (A allele)	28 (43%)	21 (30%)	0.17
−12669C>T (rs1042031) (T allele)	17 (26%)	16 (23%)	0.84
ACE I/D rs1799752 (D allele)	36 (56%)	39 (52%)	0.96

Data are reported as means±SD, except where indicated. n: number of patients; SBP: systolic blood pressure; DBP: diastolic blood pressure; HR: heart rate at rest; bpm: beats per minute; MI: myocardial infarction; PCI: percutaneous coronary intervention; CABG: coronary artery bypass graft; ACE: angiotensin converting enzyme. *t*-tests were used to compare age and hemodynamic variables and the chi-squared test was applied for categorical variables.

Table 2 shows lipid profile and cardiorespiratory variables obtained at VAT. No significant group × time interaction was observed. However, after 16 weeks, apolipoprotein B levels decreased (main time effect; F1.62=5.20; P=0.02), while LDL levels increased (main time effect; F1.62=6.98; P=0.01) in both groups.

**Table 2.** Lipid profile and cardiorespiratory variables of the groups studied at baseline and after 16 weeks.

	Trained (n=32)			Control (n=34)		
	Baseline	Post-16	Mean difference (95%CI)	Baseline	Post-16	Mean difference (95%CI)
**Lipid profiles**						
TG (mg/dL)	127.56±62.33	111.44±56.34	−16.12 (−45.22; 12.97)	165.73±103.87	141.66±60.05	−25.13 (−44.74; 13.65)
TC (mg/dL)	169.81±41.88	176.38±41.52	6.57 (−9.98; 22.50)	172.88±38.05	170.84±34.23	−2.04 (−17.37; 12.86)
HDL-C (mg/dL)	44.63±14.87	46.09±12.32	1.46 (−0.56; 6.40)	40.71±9.24	42.12±11.70	1.41 (−1.94; 4.54)
LDL-C (mg/dL)	95.88±30.54	114.58±34.01	18.70 (−0.30; 34.64)	93.21±38.22	103.63±30.07	10.42 (−7.57; 24.95)
ApoA1 (mg/dL)	138.28±18.48	137.93±16.33	−0.35 (−7.64; 7.04)	127.94±18.36	126.27±19.40	-1.67 (−8.75; 4.91)
ApoB (mg/dL)	96.48±27.63	87.49±20.19	−8.99 (−21.35; −2.05)	98.06±26.44	93.03±19.74	−5.03 (−15.33; 2.63)
**Ventilatory anaerobic threshold**						
VO_2_ (mL^.^kg^-1.^min^-1^)	13.59±3.18	15.53±3.46***^+^	2.05 (1.10; 3.00)	12.84±2.73	11.27±2.55**	−1.57 (−2.58; −0.68)
VO_2_ (L/min)	1.09±0.24	1.24±0.32***^+^	0.16 (0.08; 0.24)	1.05±0.24	0.93 ±0.24**	−0.12 (−0.20; −0.04)
HR (bpm)	103.31±15.96	104.34±16.20	1.25 (−3.39; 5.89)	103.50±21.32	96.72±16.70**	−6.78 (−11.70; −2.41)
Workload (W)	80.16±24.04	98.53±26.22***^+^	19.06 (11.52; 26.60)	80.44±20.52	72.41±19.03*	−8.03 (−15.38; −0.30)
SBP (mmHg)	167.84±19.67	166.78±16.41	−0.75 (−6.49; 4.99)	161.35±21.64	163.00±21.28	1.65 (−5.12; 6.37)
DBP (mmHg)	91.56±10.22	89.53±9.87	−2.03 (−5.47; 1.41)	93.65±12.57	94.28±10.44	−0.63 (−3.66; 3.22)

Data are reported as means±SD (Baseline and Post-16); Mean difference (Post-16 values minus Baseline values) with 95% of confidence interval (CI); TG: triglycerides; TC: total cholesterol; HDL-C: high-density lipoprotein cholesterol; LDL-C: low-density lipoprotein cholesterol; ApoA1: apolipoprotein A1; ApoB: apolipoprotein B; VO_2_: oxygen consumption; VCO_2_: carbon dioxide production; W: watts; HR: heart rate; bpm: beats per minute; SBP: systolic blood pressure; DBP: diastolic blood pressure. *P<0.05, Baseline *vs* Post-16; **P<0.01, Baseline *vs* Post-16; ***P<0.001, Baseline *vs* Post-16; ^+^P<0.05, Baseline (trained) *vs* Baseline (control); Post-16 (trained) *vs* Post-16 (control) (two-way ANOVA).

Cardiorespiratory variables obtained at the VAT showed significant group × time interaction for VO_2VAT_ (F1.62=30.21, P<0.001), workload (F1.62=25.43, P<0.001), and HR_VAT_ (F1.62=6.40, P=0.01). Planned pairwise comparisons revealed a significant increase in workload and VO_2VAT_ for the trained group after the AIT (P<0.05). On the other hand, control group showed a significant reduction of VO_2VAT_, workload, and HR_VAT_ (P<0.05). In addition, the trained group showed higher VO_2VAT_ and workload compared to control group after the AIT (P<0.05).

Lipid profiles at baseline and after 16 weeks according to *ApoB* polymorphisms are presented in Table 3 (12669C>T – rs1042031) and Table 4 (−7673G>A – rs693). Considering the small number of patients with TT genotype (−12669C>T), these patients were combined with CT genotype (−12669C>T) (co-dominant model) and AA genotype (−7673G>A) was combined with GA genotype (−7673G>A) (dominant model).

**Table 3.** Baseline and post-16 weeks lipid profile of trained and control groups according to −12669C>T (rs1042031) in the *ApoB* gene.

	Trained (n=32)			Control (n=34)			Total (n=66)
	Baseline	Post-16	Mean difference (95%CI)	Baseline	Post-16	Mean difference (95%CI)	Baseline
**CC**	**n=17**			**n=19**			**n=36**
TG (mg/dL)	111.12±66.94	105.18±60.24	−5.97 (−32.21; 44.09)	166.28±109.54	153.59±72.42	−12.69 (−56.80; 19.50)	139.49±94.29
TC (mg/dL)	172.82±46.11	188.12±47.25	15.29 (−4.02; 34.61)	175.06±40.50	178.06±28.91	3.00 (−17.96; 20.67)	175.17±42.68
HDL-C (mg/dL)	47.35±18.25	47.29±14.36	−0.059 (−4.38; 4.50)	40.56±9.10	41.93±10.94	1.37 (−3.51; 5.37)	43.92±14.29
LDL-C (mg/dL)	95.82±33.48	123.76±41.30	27.93 (6.92; 48.94)	96.63±38.39	107.11±22.64	10.48 (−10.27; 31.74)	96.10±36.08
ApoA1 (mg/dL)	146.53±17.24	145.69±14.91	−0.84 (−9.79; 8.11)	125.78±18.01	129.44±21.31	3.66 (−6.28; 11.62)	135.53±20.12
ApoB (mg/dL)	94.11±27.81	90.10±20.51	−4.00 (−15.89; 7.88)	95.20±22.30	94.32±19.35	−0.88 (−14.54; 9.23)	95.07±24.52
**CT+TT**	**n=13+2**			**n=13+2**			**n=30**
TG (mg/dL)	146.20±52.73	118.53±52.74	−27.99 (−69.68; 12.95)	165.07±100.44	131.71±39.25	−33.36 (−50.55; 36.70)	155.63±79.40
TC (mg/dL)	166.40±37.81	163.07±30.16	−3.33 (−23.89; 17.32)	165.33±35.25	164.14±39.82	−1.19 (−28.70; 15.47)	166.87±35.92
HDL-C (mg/dL)	41.53±9.45	44.73±9.84	3.20 (−1.53; 7.93)	40.53±9.92	42.93±13.16	2.4 (−3.46; 6.69)	41.03±9.53
LDL-C (mg/dL)	95.95±28.00	104.17±19.85	8.22 (−14.13; 30.58)	89.11±38.95	100.16±39.11	11.05 (−18.67; 29.36)	92.53±33.51
ApoA1 (mg/dL)	126.58±15.86	129.15±13.44	2.56 (−6.96; 12.10)	130.80±19.61	123.09±17.55	−7.71 (−18.17; 2.31)	128.69±17.65
ApoB (mg/dL)	96.53±29.26	84.53±20.12	−12.00 (−24.66; 0.65)	100.77±31.89	92.54±21.18	−8.23 (−24.04; 2.79)	98.65±30.15

Data are reported as means±SD (Baseline and Post-16). Mean difference (Post-16 values minus Baseline) with 95% confidence interval (CI) (three-way ANOVA); TG: triglycerides; CT: total cholesterol; HDL-C: high-density lipoprotein cholesterol; LDL-C: low-density lipoprotein cholesterol; ApoA1: apolipoprotein A1; ApoB: apolipoprotein B.

No significant group × genotype × time interactions were observed for any of the study variables for 12669C>T of the *ApoB* gene (P>0.05) (Table 3). However, after 16 weeks, LDL levels significantly increased (main time effect; F_1.62_=6.68, P=0.01) in both control and trained groups and in CC and CT+TT genotypes. Moreover, triglyceride levels were higher in the control group than in the trained group in both genotypes (CC and CT+TT) at baseline and after 16 weeks (main group effect; F_1.62_=4.54, P=0.01).

Regarding the polymorphism −7673G>A of the *ApoB* gene (Table 4), there was significant genotype × time interaction for the LDL levels (F_1.62_=4.25; P=0.04). After 16 weeks, control and trained groups with GA+AA genotype presented significantly increased LDL levels (P<0.05). Significant group × genotype interaction for LDL (F_1.62_=4.05; P=0.04) and apolipoprotein B levels (F_1.62_=4.00; P=0.04) was also observed. Both at baseline and after 16 weeks, LDL levels were higher in the trained group compared to the control group with GA+AA genotype (P<0.05), whereas apolipoprotein B levels were lower in the trained group compared to the control group with GG genotype (P<0.05).

**Table 4.** Baseline and post-16 weeks lipid profile of trained and control groups according to −7376G>A (rs693) genotypes of the *ApoB* gene.

Genotypes	Trained (n=32)			Control (n=34)			Total (n=6)
	Baseline	Post-16	Mean difference (95%CI)	Baseline	Post-16	Mean difference (95%CI)	Baseline
**GG**	**n=10**			**n=16**			**n=26**
TG (mg/dL)	126.80±55.69	105.00±29.30	−21.80 (−75.47; 27.90)	144.13±76.53	146.13±70.90	2.00 (−27.64; 52.44)	137.46±68.59
TC (mg/dL)	153.30±47.44	157.60±32.31	4.30 (−20.84; 33.28)	175.75±32.84	166.47±34.14	−9.28 (−28.63; 13.29)	167.12±39.77
HDL-C (mg/dL)	38.70±7.06	44.30±10.00	5.60 (−0.82; 11.26)	39.50±10.40	41.67±13.81	2.17 (−2.74; 6.61)	39.19±9.11
LDL-C (mg/dL)	91.48±34.16	102.32±32.28	10.84 (−13.84; 42.73)	108.83±26.75	101.79±29.76	−7.04 (−29.26; 14.55)	102.15±30.39
ApoA1 (mg/dL)	131.78±12.15	131.02±13.08	−0.76 (−11.79; 13.39)	127.69±17.69	123.35±17.64	−4.34 (−14.09; 5.41)	129.16±15.77
ApoB (mg/dL)	93.00±29.75	79.45±12.81	−13.55 (−1.94; 30.05)	107.05±28.68^+^	94.62±18.18^+^	−12.43 (−25.58; −0.80)	101.99±29.27
**GA + AA**	**n=16+6**			**n=15+3**			**n=40**
TG (mg/dL)	127.91±66.37	114.36±65.49	−13.54 (−46.61; 19.51)	186.06±123.20	137.71±50.54	−48.35 (−79.57; 0.51)	153.26±98.38
TC (mg/dL)	177.32±37.89	184.91±43.06	7.59 (−9.72; 24.90)	170.33±42.94	174.71±34.88	4.38 (−17.49; 24.43)	174.18±39.86
HDL-C (mg/dL)	47.32±16.75	46.91±13.38	−0.49 (−4.27; 3.45)	41.78±8.22	42.52±9.88	0.74 (−4.16; 5.20)	44.83±13.72
LDL-C (mg/dL)	97.88±29.38^+^	120.15±34.01*^+^	22.26 (4.17; 40.36)	78.52±42.17	105.34±31.22*	26.82 (2.24; 46.06)	89.44±36.34
ApoA1 (mg/dL)	140.94±20.15	141.08±16.95	0.13 (−7.92; 8.19)	128.17±19.44	128.85±21.03	0.68 (−9.26; 10.25)	135.19±20.61
ApoB (mg/dL)	97.90±27.31	91.14±22.07	−6.75 (−16.99; 3.47)	90.08±22.08	91.63±21.48	1.55 (−11.57; 13.21)	94.38±25.09

Data are reported as means±SD (Baseline and Post-16). Mean difference (Post-16 values minus Baseline) with 95% confidence interval (CI); TG: triglycerides; CT: total cholesterol; HDL-C: high-density lipoprotein cholesterol; LDL-C: low-density lipoprotein cholesterol; ApoA1: apolipoprotein A1; ApoB: apolipoprotein B. *P< 0.05, baseline *vs* Post-16; ^+^P<0.05, baseline (trained) *vs* baseline (control) and Post-16 (trained) *vs* Post-16 (control) (three-way ANOVA).

Furthermore, at baseline and after 16 weeks, triglyceride levels (main group effect; F_1.62_=4:53, P=0.03) were higher in the control group than in the trained group, while apolipoprotein A1 levels (main group effect; F_1.62_=4.88, P=0.03) were higher in the trained group compared to the control in both genotypes (GG and GA+AA).

Cardiorespiratory variables obtained at VAT, baseline, and after 16 weeks, according to *ACE I/D* polymorphisms are presented in Table 5. There was a significant group × genotype×time interaction for VO_2VAT_ (F_1.62_=4.387, P=0.01), power output (F_1.62_=6.801, P=0.002), and HR_VAT_ (F_1.62_=3.890, P=0.02). After the AIT program, VO_2VAT_ and power output significantly increased in the trained group with *ACE* II and ID genotypes, while for the trained group with *ACE* DD genotype, variables did not differ significantly after AIT (P>0.05). The control group with *ACE* II and DD genotypes showed a significant decrease in VO_2VAT_ and HR_VAT_ (P<0.05). Furthermore, the trained group with *ACE* II genotype showed greater increase in VO_2VAT_ and power output in response to AIT, compared to the other genotypes (P<0.05).

**Table 5.** Cardiorespiratory variables obtained at ventilatory anaerobic threshold in baseline and Post-16 weeks of trained and control groups according to *ACE I/D* (rs1799752) genotypes.

Genotypes	Trained (n=32)			Control (n=34)			Total (n=66)
	Baseline	Post-16	Mean difference (95%CI)	Baseline	Post-16	Mean difference (95%CI)	Baseline
**II**	**n=6**			**n=7**			**n=13**
VO_2_ (mL^.^kg^−1.^min^−1^)	12.65±5.62	17.07±5.70***^+^	4.41 (2.32; 6.51)^#^	13.60±2.77	11.23±2.43**	−2.37 (−4.79; −0.60)	13.16±4.15
VO_2_ (L/min)	1.09±0.30	1.46±0.35***^+^	0.37 (0.19; 0.55)	1.09±0.20	0.89±0.24**	−0.20 (−0.38; −0.02)	1.09±0.24
HR (bpm)	104.17±18.84	114.00±15.48	9.83 (−0.32; 19.99)	105.14±21.17	95.33±18.37**	−9.81 (−24.32; −4.00)	104.69±19.29
Workload (W)	70.00±32.54	117.83±23.95***^+^	47.83 (32.11; 63.55)^#∞^	80.43±16.67	62.83±16.08	−17.60 (−30.71; 0.71)	75.62±24.69
SBP (mmHg)	175.00±20.74	178.33±9.83	3.33 (−9.93; 6.60)	164.29±15.92	165.00±20.74	0.71 (−16.60; 9.93)	169.23±18.35
DBP (mmHg)	97.50±11.73	101.57±7.53	4.16 (−3.56; 11.89)	96.43±16.51	93.33±10.33	−3.10 (−15.22; 0.22)	96.92±13.93
**ID**	**n=20**			**n=15**			**n=35**
VO_2_ (mL^.^kg^−1.^min^−1^)	13.51±2.55	15.45±3.21**^+^	1.42 (0.27; 2.57)	11.67±2.22	11.00±2.75	−0.67 (−2.00; 0.73)	12.79±2.48
VO_2_ (L/min)	1.11±0.26	1.26±0.31*^+^	0.10 (0.005; 0.20)	1.00±0.29	0.93±0.26	−0.07 (−0.19; 0.04)	1.06±0.26
HR (bpm)	105.82±15.97	105.94±15.45	0.20 (−5.36; 5.76)	99.87±22.49	98.07±20.19	−1.80 (−7.07; 6.22)	101.26±19.27
Workload (W)	82.24±23.52	98.18±28.05*^+^	12.25 (3.64; 20.85)	79.13±24.78	76.57±21.53	−2.56 (−13.43; 7.14)	81.71±22.97
SBP (mmHg)	173.00±20.11	168.65±15.41	−2.55 (−9.81; 4.71)	157.27±26.43	164.50±26.61	7.23 (−1.97; 15.40)	164.06±23.87
DBP (mmHg)	94.41±8.15	89.71±8.38	−4.00 (−8.23; 0.23)	91.33±11.90	93.50±11.57	2.17 (−2.98; 7.13)	91.86±10.54
**DD**	**n=6**			**n=12**			**n=18**
VO_2_ (mL^.^kg^−1.^min^−1^)	14.42±2.74	15.57±0.55^+^	1.81 (−0.27; 3.91)	13.88±2.92	11.61±2.56**	−2.27 (−3.74; −0.78)	14.06±2.79
VO_2_ (L/min)	1.05±0.24	1.12±0.22	0.13 (−0.04; 0.31)	1.09±0.22	0.94±0.23*	−0.15 (−0.27; −0.02)	1.07±0.22
HR (bpm)	105.83±10.63	100.83±11.97	−3.83 (−13.99; 6.32)	107.08±21.01	95.83±12.09**	−11.25 (−18.43; −4.06)	106.67±17.87
Workload (W)	78.67±22.71	88.00±16.22	13.00 (−2.71; 28.71)	82.08±18.03	72.33±16.82	−9.75 (−20.86; 1.36)	80.94±19.10
SBP (mmHg)	156.33±8.04	155.83±14.97	1.16 (−12.10; 14.43)	164.75±18.34	160.25±15.19	−4.50 (−13.88; 4.88)	161.94±15.91
DBP (mmHg)	83.33±5.16	81.67±4.08	−1.66 (−9.39; 6.06)	94.92±11.46	95.67±9.86	0.75 (−4.71; 6.21)	91.06±11.15

Data are reported as means±SD (Baseline and Post-16). Mean difference (Post-16 values minus Baseline values) with 95% confidence interval (CI). ACE: angiotensin converting enzyme; I/D: insertion/deletion; VO_2_: oxygen consumption; VCO_2_: carbon dioxide production; W: watts; HR: heart rate; bpm: beats per minute; SBP: systolic blood pressure; DBP: diastolic blood pressure. *P<0.05, Baseline *vs* Post-16; **P<0.01, Baseline *vs* Post-16; ***P<0.001, Baseline *vs* Post-16; ^+^P<0.05, Baseline (trained) *vs* Baseline (control); Post-16 (trained) *vs* Post-16 (control); ^#^P<0.05, II *vs* ID genotypes; ^∞^P<0.05, II *vs* ID and DD genotypes (three-way ANOVA).

**Figure 2.**
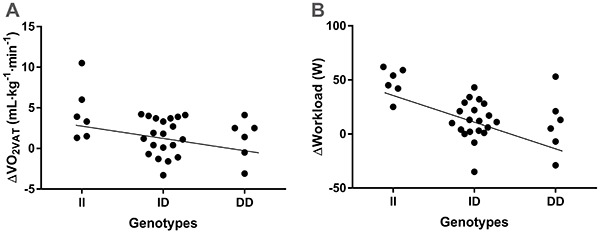
Change in oxygen uptake (VO_2_) (*A*) and power output (*B*) at the ventilatory anaerobic threshold according to angiotensin-converting enzyme I/D (rs1799752) genotypes. I/D: insertion/deletion.

In the trained group, changes in VO_2_ (r=-0.36; P=0.03: Figure 2A) and power output (r=-0.52, P=0.003: Figure 2B) at VAT were correlated with the *ACE I/D* polymorphism.

## Discussion

The present study showed that trained and control groups with polymorphism −7376G>A (rs693) of the *ApoB* gene presented increased LDL-C levels after 16 weeks. After the AIT program, the increased functional aerobic capacity in the trained group was associated with the *ACE* I-allele (rs1799752).

To our knowledge, this was the first study that provided evidence of changes in the lipid profile in relation to polymorphisms −7376G>A and −12669C>T of the *ApoB* gene in response to an AIT program in patients with CAD and/or risk factors. However, serum lipid response was significantly different only in the presence of polymorphism −7376G>A in both trained and control groups. Exercise training promotes reduction in LDL levels, however, in this study LDL levels increased in trained and control groups with the GA+AA genotype after 16 weeks. Although we found no rational explanation for this result, it is possible that the lack of dietary intake control may have impaired the detection of exercise-induced changes in the lipid profile. To overcome these practical difficulties, measurements of the main apolipoprotein moieties of LDL, i.e. apolipoprotein B, have been proposed to serve as alternative surrogate markers. The molar amount of apolipoprotein B in whole serum has the desirable property of being an estimate of total LDL-C particles, since there is one apolipoprotein B molecule per LDL particle (26). In this study, changes in apolipoprotein B levels were lower and LDL levels were higher in trained and control group with GA+AA genotype. In this case, monitoring LDL-C to quantify improvements in the lipid profile should be replaced by apolipoprotein B when exercise is used for cardiovascular risk reduction.

The polymorphism −7376G>A of the *ApoB* gene is known to be a silent mutation. Thus, a change does not alter the amino acid sequence of apolipoprotein B. Considering that this polymorphism cannot directly compromise lipid metabolism, Chiodini et al. (10) and Boekholdt et al. (27) suggested that it may be in linkage disequilibrium with other variants in the *ApoB* gene itself or nearby, affecting the LDL-receptor-binding region of apolipoprotein B. This linkage disequilibrium has been reported by Machado et al. (9) between two *ApoB* polymorphisms [(*Ins/Del* − Insertion/Deletion (rs17240441) and *XbaI* − X+/X- (rs693)] in a Brazilian population. These authors observed that haplotypes formed by X+ and Del alleles (X+Del haplotype) were associated with significantly higher serum levels of total cholesterol, triglycerides, and LDL-cholesterol. In this specific case, a single haplotype, X+Del may contribute to structural changes that occur in apolipoprotein B100 caused by this variation, compromising the interaction between apolipoproteins and LDL receptors, leading to the accumulation of LDL in circulation (9,10,27,28).

In relation to the −12669C>T polymorphism, the exchange of cytosine by thymine leads to amino acid sequence substitution, which may affect the tertiary structure of apolipoprotein B and its interaction with LDL. Several studies have shown clear evidence of the association between polymorphisms of the ApoB gene and changes in lipid profile, specifically with LDL (10,27,29,30). However, studies evaluating the functionality of structural domains of the apolipoprotein B100 protein highlighted that this mutation occurs in amino acid 4154 or after the binding site, between amino acids 3130 and 3630, which potentially interacts with the LDL receptor (28–30). This fact would explain the absence of significant changes in LDL levels in this polymorphism.

This study showed that VO_2VAT_ and power output at baseline were similar among I/D genotypes of the *ACE* gene. After AIT, the increase in VO_2VAT_ and power output were observed in the presence of the *ACE* I-allele. In addition, the trained group with *ACE* II genotype showed greater adaptive responses, which was supported by the greater increase in VO_2VAT_ and power in the group with *ACE* ID genotype.

Previous studies involving healthy subjects have shown clear evidence that the *ACE* I-allele is associated with better aerobic performance as well as with better response to aerobic training (12,13). However, in individuals with heart disease, studies are still controversial in relation to the *ACE* I-allele, and changes in the VO_2max_ are dependent on this allele (11,31). Moreover, these studies were based on the increase in VO_2max_, which for cardiac patients is a difficult parameter to define maximum performance. In this study, aerobic functional capacity was evaluated from cardiorespiratory parameters obtained at VAT during the submaximal stress test. Therefore, our results showed that the *ACE* I-allele can be an important modulator in the increase of VO_2_ and power at VAT.

Considering that *ACE* is part of the renin-angiotensin system, which can be found in the cardiovascular system as well as in several other tissues, some physiological mechanisms have been suggested to explain the causal relationship in the greater adaptive response attributed to the *ACE* I-allele. One of these mechanisms considers that a lower amount of circulating ACE may reduce bradykinin degradation and increase bioavailability of nitric oxide, thus improving the efficiency of mitochondrial respiration and adjusting the local regulation of the aerobic metabolism (11,32). Moreover, Zhang et al. (33) found that the presence of the *ACE* I-allele may be associated with an increased percentage of type I fibers in skeletal muscles, which allows for greater aerobic performance and improves mechanical and metabolic efficiency.

This study has some limitations that should be discussed. First, patients with CAD and/or cardiovascular risk factors were under treatment with beta-blockers and ACE inhibitors, which could affect circulating ACE levels. The same could occur with patients treated with statins. Second, the sample size was relatively small to conduct a genetic study, which may require more statistical power to explore the real association. However, this pilot study evaluated the possibility of continuing the study in the near future. Third, patients were randomized into trained and control groups, which makes an even distribution among polymorphisms difficult. Finally, the possibility that some other genetic factor associated with *ApoB* variants is responsible for differences in the lipid profile cannot be ruled out.

In conclusion, the presence of allele I of the *ACE* gene was associated with increased aerobic functional capacity after the AIT program. Regarding the lipid profile, LDL levels increased after 16 weeks in patients with polymorphism −7673G>A of the *ApoB* gene. Thus, these results provided a partial explanation for the variability of AIT adaptive responses in patients with CAD and/or cardiovascular risk factors.

This pilot study has demonstrated that the variability of the adaptive responses in the lipid profile and functional capacity with physical training may be attributed to genetic polymorphisms. *ACE* and *ApoB* polymorphisms are associated with this inter-individual variability. Although the mechanisms and hypotheses involved in the synthesis of structural proteins and enzymes are relatively clear, this is the first study that demonstrated evidence of changes in the lipid profile in relation to polymorphisms −7376G>A and −12669C>T of the *ApoB* gene, as well as changes in functional capacity in relation to allele I of the *ACE* gene. These findings are important to identify response and no-response individuals submitted to cardiac rehabilitation.

## Supplementary Material

Click here to view [pdf]
